# Long-lasting microbubble-enhanced super-resolution ultrasound imaging unveils lymphatic activity in lymph node

**DOI:** 10.7150/thno.117971

**Published:** 2025-08-11

**Authors:** Jingyi Yin, Feihong Dong, Xiangui Zhang, Yuan Peng, Pengting Min, Jiabin Zhang, Shu Wang, Jing Fang, Jue Zhang

**Affiliations:** 1 Academy for Advanced Interdisciplinary Studies, Peking University, Beijing, China.; 2 College of Future Technology, State Key Laboratory of Membrane Biology, Peking-Tsinghua Center for Life Sciences, and Institute of Molecular Medicine, Peking University, Beijing, China.; 3 National Biomedical Imaging Center, Peking University, Beijing, China.; 4 Breast Disease Center, Peking University People's Hospital, Beijing, China.; 5 New Cornerstone Science Laboratory, CAS Key Laboratory of Biomedical Effects of Nanomaterials and Nanosafety and CAS Center for Excellence in Nanoscience, National Center for Nanoscience and Technology of China, Beijing, China.; 6 College of Engineering, Peking University, Beijing, China.

**Keywords:** Super-resolution ultrasound imaging, Microbubble contrast agent, Lymph node imaging, Multiparametric hemodynamic imaging, Functional vascular phenotyping

## Abstract

**Rationale:** As the central regulatory hub localized at the interfaces between the blood and lymphatic vascular system, lymph nodes play a pivotal role in maintaining immune homeostasis and coordinating responses to disease. Systematic evaluation of lymph node functional status holds significant clinical value. However, there are currently no established non-invasive imaging biomarkers capable of reliably evaluating lymph node activity.

**Methods:** This study developed a novel lipid-based long-lasting microbubble contrast agent (SuperVue-MB) integrated with three-dimensional super-resolution ultrasound (SRUS) imaging to achieve multiparametric hemodynamic visualization of blood vessels within lymph nodes.

**Results:** Experimental validation demonstrated strong correlations between SRUS-derived vascular parameters and established reference standards (*r* = 0.86, p < 0.01), including micro-CT angiography and histopathological markers, confirming the reliability and accuracy of the technique. Furthermore, this study introduces a new vascular subtyping strategy that systematically correlates these hemodynamic patterns with immune functional states. Specifically, the density and number of microbubble tracks associated with key vascular subtypes, exhibiting significant positive correlations with lymphatic vessel density (*r* = 0.91, p < 0.01).

**Conclusions:** This study offers a promising noninvasive approach for evaluating immune activation within lymph nodes, while establishing a novel paradigm for surveillance of sentinel lymph node metastasis and immunotherapy response monitoring.

## Introduction

Lymph nodes (LNs) play a vital role in immune surveillance, maintaining physiological homeostasis, and facilitating immune defense 1, 2. Within LNs, two distinct but interrelated vascular systems, the blood vasculature and the lymphatic vasculature, coordinate to regulate immune responses 3. Alterations in lymphatic vessel architecture can influence tumor-associated angiogenesis, while inflammation-induced vascular permeability within the blood microvasculature can promote both lymphangiogenesis and edema formation 3, 4. Notably, the blood vessels, especially microvessels of LNs, facilitate immune cell trafficking and lymphatic drainage 5, 6, with dysfunction implicated in conditions such as cancer, lymphedema, and inflammatory disorders. Therefore, comprehensive characterization of both LN blood microcirculation and lymphatic vessel networks holds significant promise for advancing our understanding of immune dynamics and guiding therapeutic interventions.

Traditionally, clinical evaluation of LNs relies on invasive procedures such as LN dissection and sentinel lymph node biopsy. While informative, these methods carry risks of complications, such as infection and lymphedema, and impose considerable economic burdens. This underscores the need for noninvasive imaging techniques that can accurately characterize LN pathology.

Contrast-enhanced ultrasound (CEUS) is a promising noninvasive imaging tool that enables real-time visualization of blood flow perfusion. It has been successfully used to identify sentinel LNs and differentiate between benign and malignant LNs 7. For example, Kodama et al. demonstrated that high-resolution CEUS could detect perfusion deficits in metastatic LNs of MRL/lpr mice  8. However, benign conditions such as inflammation and infection may mimic the vascular patterns observed in malignancies, and small malignant LNs may not show obvious perfusion changes, leading to potential misclassification.

Super-resolution ultrasound (SRUS) imaging overcomes the spatial resolution limitations of conventional CEUS by tracking moving microbubbles (MBs) within the blood vessels. This technique enables noninvasive, high-resolution visualization of microvascular architecture and function, and has been applied successfully to various organs 9-14, including the brain, kidneys, and heart  15-18. Using a multi-plane scanning platform, Zhu et al. successfully achieved 3D imaging of microvasculature in rabbit LNs 19. Moreover, distinct blood flow patterns between normal and metastatic LNs have been observed 20, further underscoring the potential of SRUS for LN evaluation.

Despite these advances, current applications of SRUS remain largely focused on quantifying global blood vasculature, with limited attention to the structural and functional heterogeneity of the dense vascular networks within LNs. To date, no noninvasive imaging method has been established to characterize the interplay between lymphatic and blood vessels within LNs.

Furthermore, SRUS faces several technical challenges. Conventional ultrasound localization microscopy (ULM) requires extended acquisition times, while commercially available MBs exhibit short circulation durations *in vivo*. Although infusion-based protocols can prolong MB persistence, they are technically demanding. These limitations highlight the need for stable, long-circulating contrast agents to enhance the feasibility, reliability, and translational applicability of SRUS in clinical and research settings.

To address these challenges, we developed a homogenization-based method for large-scale production of high-stability long-lasting ultrasound MBs (SuperVue-MB). Using MRL/lpr mice, a well-established autoimmune model, we integrated micro-computed tomography (micro-CT), SRUS imaging, and immunofluorescence-based lymphatic visualization to investigate the microvascular-lymphatic architecture of LNs. Moreover, we developed a new vascular subtyping strategy to explore the correlations between different hemodynamic patterns and immune functional states. Our goal is to establish a noninvasive and high-resolution imaging framework for evaluating LN immune activity, facilitating future immunopathological assessments.

## Methods and Materials

### Microbubble fabrication

Homogenization and lyophilization method were employed to fabricate MB freeze-dried powder in our study. Briefly, an amount of DSPC, DSPE-PEG2000, and palmitic acid were dissolved completely in cyclooctane to obtain solution S1; PEG4000 was dissolved completely in pure water to obtain solution S2. And then, solutions S1 and S2 was mixed and homogenized at 18,000 rpm for 5 min to obtain oil in water droplets; Then the above milk drops are transferred to a penicillin bottle for vacuum freeze drying to obtain freeze-dried powder. After freeze-drying, the upper and middle layers of the penicillin bottle are replaced with perfluoropropane, and then plugged, capped and stored at room temperature; When in use, dissolve in physiological saline to obtain long-lasting MBs (SuperVue-MB).

### Microbubble characterization

After reconstitution with physiological saline, the MB concentration was accurately calculated using a hemocytometer. The MB microstructure was captured using an inverted fluorescence microscope. The particle size and distribution of MBs were detected using a Beckman Multisizer 4e instrument. Details parameters of the measurements can be found in the literature 21.

### Animal model

All animal experimental protocols were reviewed and approved by the local animal care committee of Peking University (AAIS-ZhangJ-9).

As a well-established autoimmune mouse model, MRL/MpJ-lpr/lpr (MRL/lpr) mice have been extensively studied for their pathological changes in LNs 12, 22, 23. These mice typically exhibit spontaneous lymphoproliferation and autoantibody production 24, leading to lymphadenopathy, vascular dilation resembling immune-induced changes 22, 25, 26, and abnormal germinal center hyperplasia 27.

In this study, MRL/lpr mice (aged 13-20 weeks) were obtained from Jiangsu Huachuang Xinnuo Pharmaceutical Technology Co., Ltd. (Jiangsu, China). As the autoimmune phenotype progresses, the proper axillary lymph node (PALN) of MRL/lpr mice gradually enlarges. By 16 weeks of age, their size typically reached 8-10 mm, comparable in size to human LNs 23, 28. The mean longitudinal diameter of PALNs, measured with a digital caliper after surgical excision, was 8.57 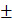
1.52 mm.

PALNs from mice of different ages were evaluated using SRUS imaging, micro-CT imaging, and histopathological analyses. A total of 11 mice were included in this study. Among them, 3 mice were used to compare SRUS and micro-CT results for consistency in vascular structural features. The remaining 8 mice underwent SRUS imaging followed by immunohistochemical staining to investigate correlations between SRUS-derived vascular parameters and histopathological indicators.

### Lymph node ultrasound imaging

All experimental data in this study were acquired using the Verasonics Vantage ultrasound acquisition system (Verasonics, Redmond, WA, USA). The system operates on a MATLAB-based platform and is equipped with a 128-element linear array transducer (L35-16vX), operating at a center frequency of 25 MHz. Seven plane waves with steering angles ranging from -7.5° to +7.5° were transmitted. The pulse repetition frequency (PRF) was set to 5,000 Hz, and the compounded plane wave frame rate was 500 Hz.

To obtain comprehensive blood perfusion signals in LNs, the transducer was mounted on a 3D motorized positioning system with a probe holder. A motorized translation stage (step size: 150 μm) was used to acquire multi-plane CEUS data of the mouse axillary lymph nodes. Prior to imaging, mice were anesthetized with 3% isoflurane for induction, and anesthesia was maintained with 1.5% isoflurane during ultrasound acquisition. The mice were secured on the imaging platform, and the probe was adjusted to align with the largest longitudinal cross-section of the LN. Subsequently, 100 μL of SuperVue-MB (concentration: 10⁸ bubbles/mL) was administered via the tail vein.

For large LNs, 20-30 imaging planes were acquired to ensure full coverage of blood flow signals. Each plane consisted of 1,500 ultrasound frames, corresponding to a 3s acquisition period. The raw radiofrequency (RF) data were stored on a computer hard drive for subsequent image reconstruction and SRUS processing.

In preliminary experiments, SRUS imaging quality was optimized by adjusting parameters such as MB concentration, ultrasound center frequency, mechanical index, and acquisition time.

### SRUS imaging of the blood vessels in lymph node

In this study, the raw RF data were beamformed to generate B-mode image sequences. To enhance MB signals, a singular value decomposition filter was applied. Since tissue signals remain relatively stationary, they correspond to larger singular values, whereas random noise signals correspond to smaller singular values. By applying an appropriate threshold, MB blood flow signals were effectively extracted.

To eliminate motion artifacts, we first excluded frames exhibiting significant tissue motion, by evaluating cross-correlation between consecutive B-mode frames. Frames with strong decorrelation typically indicated out-of-plane motion. Rigid motion estimation and compensation were then performed.

An initial intensity threshold was estimated to detect MB regions. The detected regions were then compared with the theoretical point spread function (PSF) based on their shape, intensity, and size. Any regions inconsistent with the PSF, typically corresponding to noise or overlapping MB signals, were discarded 29-32. Following the method described in 32-36, we measured the spatial resolution at the corresponding imaging frequency. Specifically, we sampled 10 individual MB PSFs and calculated the axial and lateral full width at half maximum (FWHM), resulting in values of 143 ± 6 μm and 173 ± 27 μm, respectively.

Using a centroid localization strategy, each MB center was pinpointed at subpixel resolution. The Hungarian tracking algorithm was subsequently employed to track MB centers frame by frame, enabling the reconstruction of continuous MB trajectories 29, 31.

By accumulating MB trajectories of long-lasting MBs, super-resolution vascular maps were reconstructed. Furthermore, super-resolution velocity maps were generated by assigning the average tracked MB velocity to the corresponding trajectory pixels 29, 32. The LN boundary was manually drawn based on B-mode images to exclude irrelevant blood vessels.

To distinguish different vascular subgroups, MB trajectories were categorized based on flow velocity. The vessel classification scheme in this study was designed to reflect the different hemodynamic characteristics of blood vessels in LN. Based on prior knowledge from murine LN studies 37, it is known that the hilum contains large arteries and veins, which branch into smaller arterioles, venules, and eventually form dense capillary networks. In this context, we hypothesized that higher flow velocity correlates with increased MB accumulation, indicating larger blood volume. A velocity threshold was then determined as the mean flow speed within regions where MB accumulation counts exceeded 2. MB trajectories with speed exceeding this threshold were categorized as large-diameter vessels with high flow speed (Type 1). The remaining low-velocity MB trajectories were further classified by flow direction: trajectories directed outward from the hilum to the LN surface were defined as Type 2 (arterial branches with low flow speed), while those directed inward toward the hilum were defined as Type 3 (venous branches with low flow speed).

All data processing was performed using MATLAB (R2020b, MathWorks, Natick, MA, USA).

### Calculation of lymph node vascular parameters

The vascular characteristics of LNs were systematically quantified using the following parameters:

Number of MB tracks: The count of valid MB trajectories obtained through tracking algorithms, which demonstrates a positive correlation with regional blood flow volume.

Vessel area: The total number of vascular pixels in the binarized super-resolution vascular map, reflecting the spatial distribution of functional vasculature.

Vessel density: The percentage of vascular pixels relative to the total number of pixels within the LN region, representing the proportion of perfused vascular area.

Mean flow speed: The average speed derived from all MB trajectories, serving as an indicator of blood perfusion efficiency.

Sum of MB flow count: The cumulative number of MBs passing through individual vascular pixels across all frames, providing a comprehensive measure of regional perfusion activity.

### *Ex vivo* micro-CT imaging

*Ex vivo* contrast-enhanced X-ray imaging was performed using a micro-computed tomography (micro-CT) scanner (Bruker Skyscan 1276, Bruker, Karlsruhe, Germany). A gelatin-based barium contrast agent was prepared according to the protocol described in previous study 38. A 40 mL solution of contrast agent contained 20 g of barium sulfate nanoparticles (nontoxic due to its insolubility; 1.0 ± 0.3 μm in size), 40,000 units of heparin (to prevent clotting), 0.16 g of Evans blue (to trace blood vessels), and 0.80 g of gelatin (to prevent washout during fixation). The mixed solution was stored at a warm place to prevent coagulation and was gently agitated prior to injection.

After CEUS imaging of the PALN, the left ventricle of the heart was perfused with phosphate-buffered saline to remove their blood, followed by perfusion with 4% paraformaldehyde solution to fix the tissue. The pre-heated contrast agent was then injected to fill blood vessels throughout the mouse, including those in LNs. Then, the mice were then placed at 4 °C for at least 2 h to solidify the contrast agent prevent vascular collapse. Subsequently, the PALN were collected and immersed in 4% paraformaldehyde for fixation.

Finally, micro-CT scanning was performed on the isolated LN samples at a resolution of 5 μm. The imaging data were analyzed and visualized using the 3D slicer and ImageJ.

### Histopathological evaluation of lymph node pathologies

Following euthanasia, subcutaneous LNs were immediately harvested and fixed in pre-chilled 4% paraformaldehyde (pH 7.4) at 4 °C for 24 h. After fixation, tissue was dehydrated, cleared, and embedded in paraffin. Serial sections (7 μm thickness) were cut along the maximal cross-section, with central sections selected for analysis.

Immunohistochemical staining was performed using an optimized triple-labeling protocol adapted from Loyd et al 37. The following primary antibodies were used: rabbit anti-LYVE-1 (GB113499, Servicebio, 1:8,000), rabbit anti-Collagen I (GB11022, Servicebio, 1:4,000), and rabbit anti-CD31 (GB113151, Servicebio, 1:2,000).

LYVE-1 is a specific marker of lymphatic endothelial cells and is widely used to quantify lymphatic vessel density (LVD) in contexts of inflammation and immune activation. In our study, the proportion of LYVE-1-positive area was used as a marker for LVD, reflecting lymphangiogenesis associated with increased immune response 39-41. CD31 identifies blood vessel endothelium, and Collagen I marks stromal cells, fibroblasts, and their secreted collagen networks. B-cell follicles were identified by collagen-deficient areas with sparse CD31 expression 37.

Fluorescence images were acquired using a Nikon Eclipse C1 microscope, with whole-slide scanning performed on a Pannoramic MIDI system (3DHISTECH, Hungary). The fluorescence labeling scheme was as follows: LYVE-1 (lymphatics, red, λex=543 nm), CD31 (vasculature, yellow, λex=628 nm), Collagen I (green, λex=494 nm), and DAPI (nuclei, blue, λex=377 nm).

Image analysis was conducted using MATLAB 2020b. The blood vessel density and LVD were quantified by first identifying CD31+ and LYVE-1+ regions. CD31 and LYVE-1 channels were binarized using Otsu's thresholding, followed by morphological opening to enhance connectivity 7. Luminal structures were identified as background regions enclosed by foreground pixels, and were filled to reconstruct complete vasculature. All analyses were manually validated to ensure only structures with intact lumina or linear morphology were included 42, 43. LYVE-1-positive areas were subtracted from CD31-positive regions to discriminate blood vessels from lymphatic vessels. Blood vessel density and LVD were quantified as the percentage of positive pixels relative to total area of the LN region.

### Data analysis

For the vascular parameters, this study utilized one-way analysis of variance (ANOVA) and Tukey's post-hoc test to compare the differences between SRUS-derived and micro-CT-derived vascular measurements. For correlation analysis between SRUS parameters and histological features, statistical significance was assessed using Pearson correlation coefficient.

To enhance the robustness of the correlation analysis, we reported not only the correlation coefficients (*r*) and p-values (P), but also the coefficient of determination (R^2^). GraphPad Prism (GraphPad Software, San Diego CA, USA) was used for statistical testing, data visualization, and figure generation.

## Results and discussions

### Introduction of SRUS imaging and analysis protocols

This study employed an integrated multimodal imaging approach, combining SRUS imaging with long-lasting MBs (SuperVue-MB), *ex vivo* micro-CT, and histopathological analysis, to systematically evaluate the relationship between blood vascular features and immune status in the axillary lymph nodes of MRL/lpr mice.

**Figure 1:**
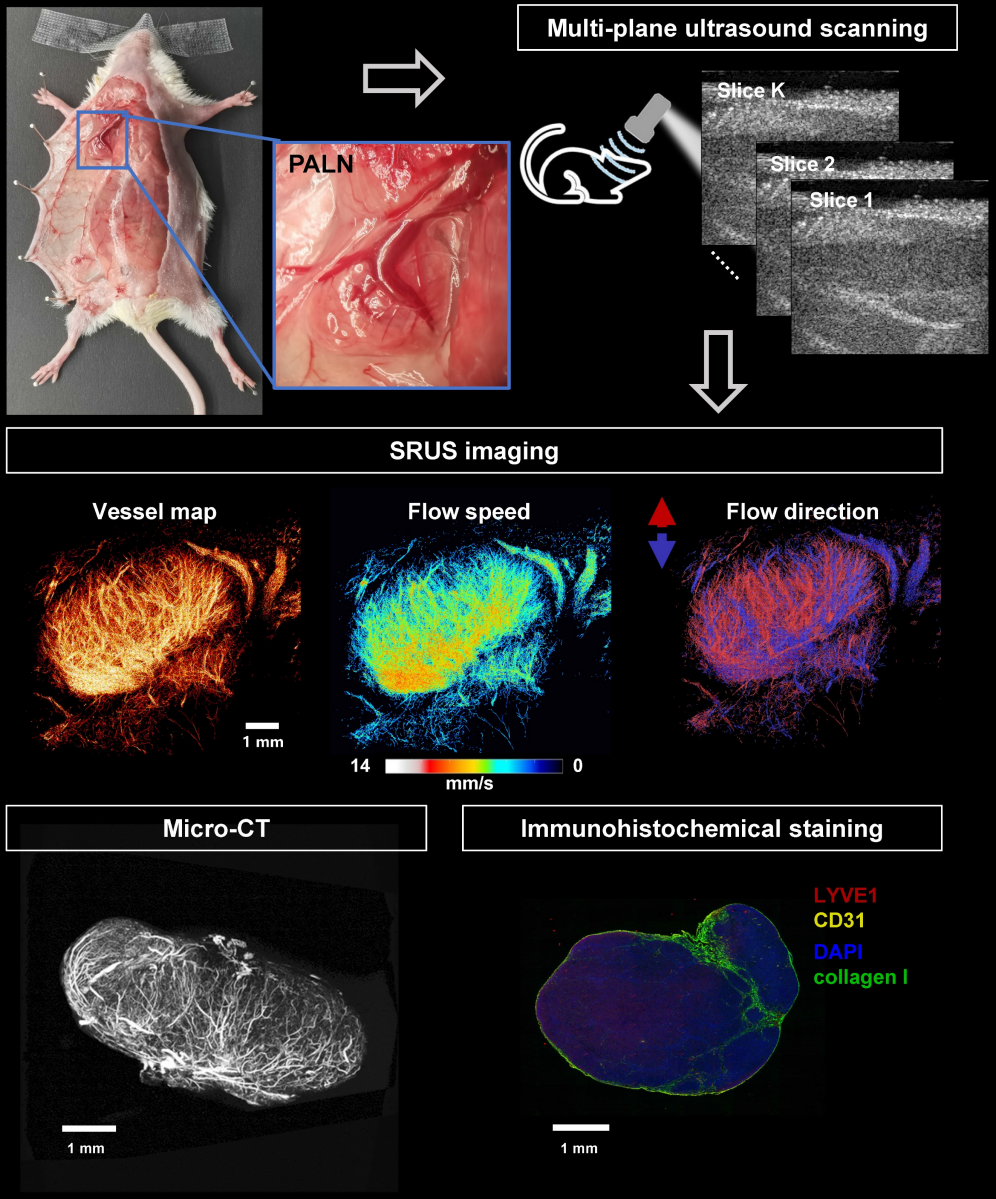
** Workflow of SRUS imaging, *ex vivo* micro-CT scanning, and immunohistochemical analysis of murine lymph nodes.** A representative photograph shows the anatomical location of the PALN in a mouse model (left panel, blue box). Multiparametric super-resolution ultrasound (SRUS) imaging was performed by multi-plane scanning (top right), followed by offline reconstruction. Three SRUS-derived vascular maps are shown, including vessel morphology (vessel map), flow speed (middle panel), and flow direction (right panel, red/blue-coded arrows for opposite axial flow directions). Validation was performed by micro-CT, which visualizes the vascular structure, and immunohistochemical staining, showing specific labeling of lymphatic vessels (LYVE1, red), blood vessels (CD31, yellow), nuclei (DAPI, blue), and collagen I (green).

The experimental workflow was illustrated in Figure 1. Initial *in vivo* SRUS imaging enabled high-resolution three-dimensional structural and functional mapping of blood vessels within the LN. This was followed by vascular perfusion with CT contrast agent for subsequent ex-vivo micro-CT imaging, allowing cross-validation of vascular structural parameters derived from SRUS and micro-CT. In addition, histopathological staining of LNs was performed to quantitatively assess angiogenic activity and to evaluate the immune status of the lymphatic tissue.

**Figure 2:**
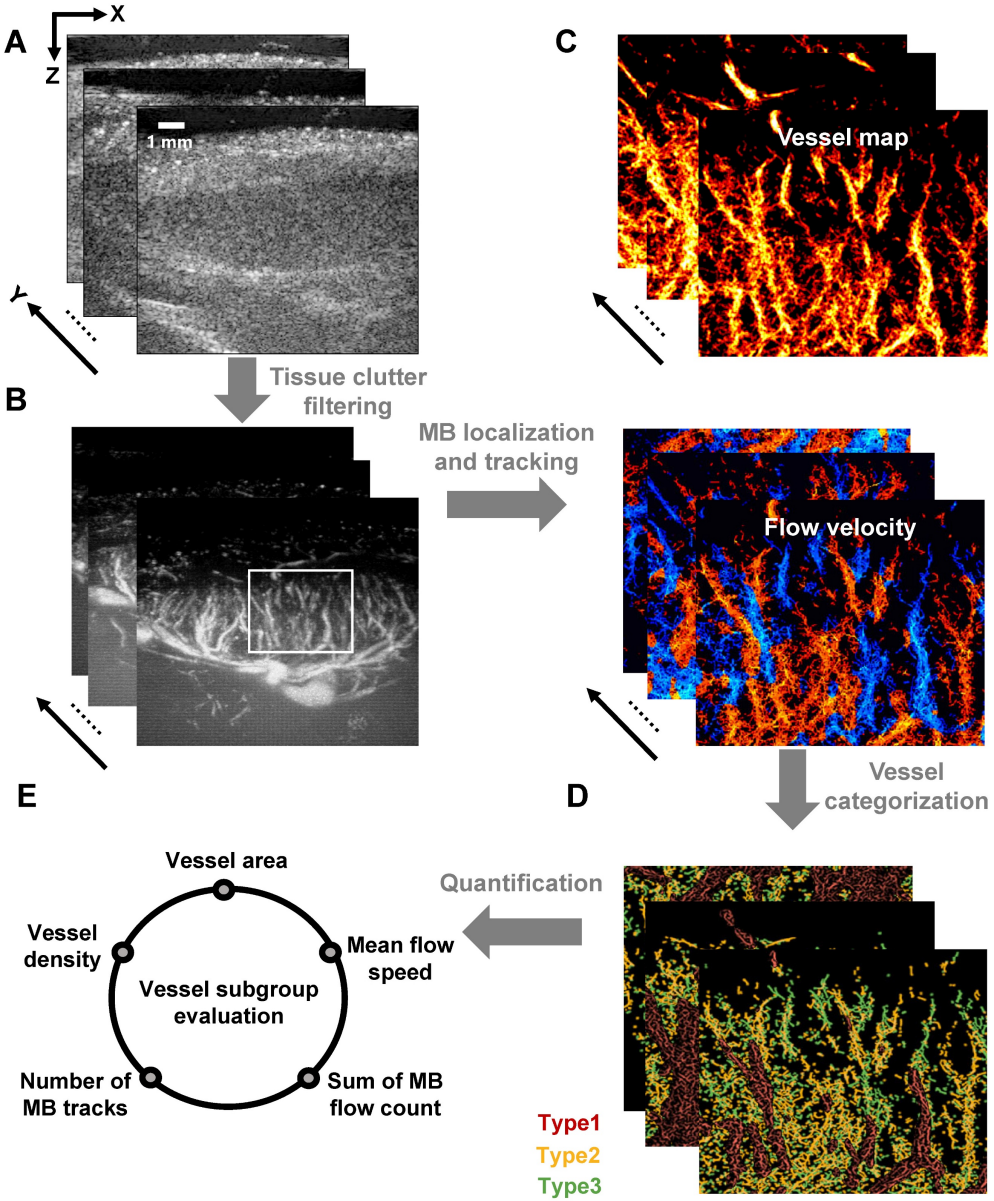
** Schematic diagram of SRUS imaging and vessel categorization strategy.** (A) Acquisition of B-mode images. (B) Extraction of flowing microbubble (MB) signals through clutter filtering. (C) Reconstructed microvascular map based on MB localization and tracking. (D) Vessel classification based on MB dynamics: Type 1 (red, large-diameter blood flow), Type 2 (yellow, low-speed arterial flow), and Type 3 (green, venous flow). (E) Quantitative characterization of microvascular parameters.

As demonstrated in Figure 2, ultrafast plane-wave imaging was employed to acquire sequential B-mode images of the LN (Figure 2A). Following data acquisition, clutter filtering was applied (Figure 2B), and microbubble localization and tracking algorithms were used to generate both super-resolution structural vascular maps and functional velocity images (Figure 2C).

To investigate the hemodynamic heterogeneity within LN blood vessels, we developed a vessel classification scheme based on the flow velocity of MB trajectories (Figure 2D). This approach categorizes blood flow into three distinct hemodynamic subtypes: high-velocity flow in large-diameter vessels, low-speed arterial flow, and low-speed venous flow. Quantitative analysis of these vascular subpopulations revealed distinct blood perfusion characteristics (Figure 2E), which not only reflect the functional status of LN vasculature but also provide a noninvasive imaging framework for the assessing immune activity within the lymphatic microenvironment.

### Characterization of microbubble contrast agents

**Figure 3:**
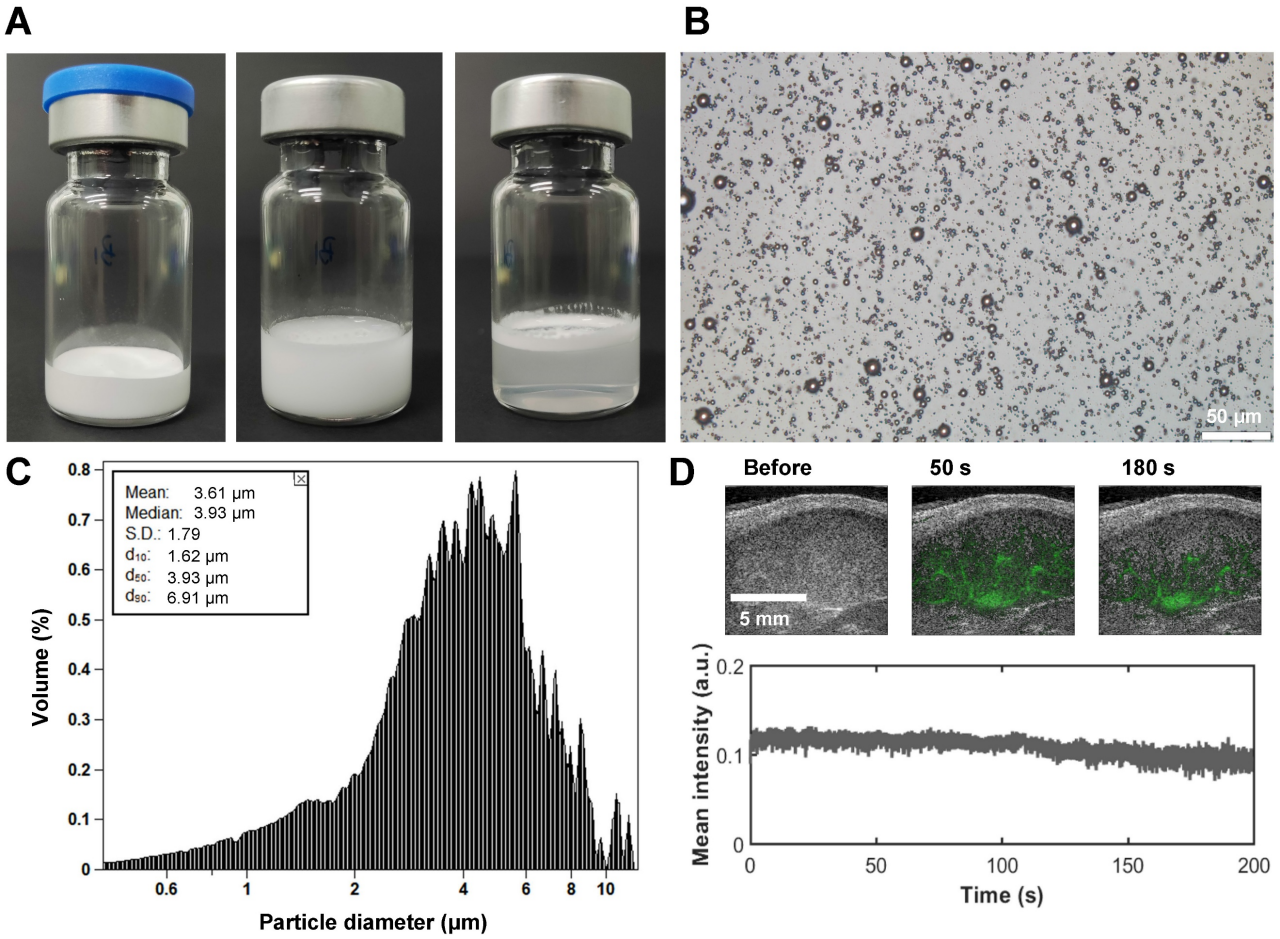
** Physicochemical characterization of SuperVue-MB.** (A) Macroscopic images of lyophilized SuperVue-MB powder and reconstituted microbubble suspension. (B) Microscopic morphology of SuperVue-MB. (C) Particle size distribution of SuperVue-MB. (D) Time-intensity curve (TIC) of SuperVue-MB contrast imaging (lower panel) in lymph nodes. Corresponding contrast-enhanced ultrasound images at selected time points (upper panel, green) are superimposed on B-mode images (grayscale) for spatial reference.

The development of ultrasound contrast agents with superior imaging performance constitutes a fundamental prerequisite for achieving stable SRUS imaging. In this study, we established an optimized preparation protocol through systematic screening of lipid MB formulations coupled with homogenization-lyophilization process optimization. This approach enabled the fabrication of contrast agents with exceptional *in vitro* stability and scalability. As shown in Figure 3A, the homogenized-lyophilized SuperVue-MBs exhibited a characteristic porous powder matrix that reconstituted into a homogeneous milky suspension upon saline rehydration. Owing to the buoyancy effect from their gaseous cores, distinct phasic separation was observed during quiescent sedimentation, with MBs accumulating at the supernatant interface to form a dense white band while maintaining optical transparency in the lower solution phase. This intrinsic physical property enables visual quality control for contrast agent concentration standardization.

Microstructural characterization (Figure 3B) revealed that the lyophilized MBs maintained spherical morphology with uniform monodispersion and minimal aggregation on microscopic examination. Size distribution analysis (Figure 3C) demonstrated narrow polydispersity and a mean diameter of 3.61 μm, falling within the optimal range for ultrasound contrast applications. *In vivo* LN imaging demonstrated rapid contrast enhancement (ΔSI >25 dB) within 1 min post-caudal vein injection, followed by sustained signal plateau persisting >3 min (Figure 3D), indicative of efficient vascular perfusion and prolonged intravascular retention, which are critical prerequisites for stable SRUS imaging. Longitudinal stability assessments (Figure S1) confirmed excellent shelf-life performance: the lyophilized powder retained original size characteristics (ΔD50 <5%) after 180-day ambient storage (Figure S1A, B), while reconstituted suspensions maintained >90% concentration stability over 24 h refrigerated (4 °C) storage (Figure S1C), meeting clinical-grade stability requirements for both long-term preservation and acute clinical deployment.

### Investigation of SRUS imaging resolution and reliability

**Figure 4:**
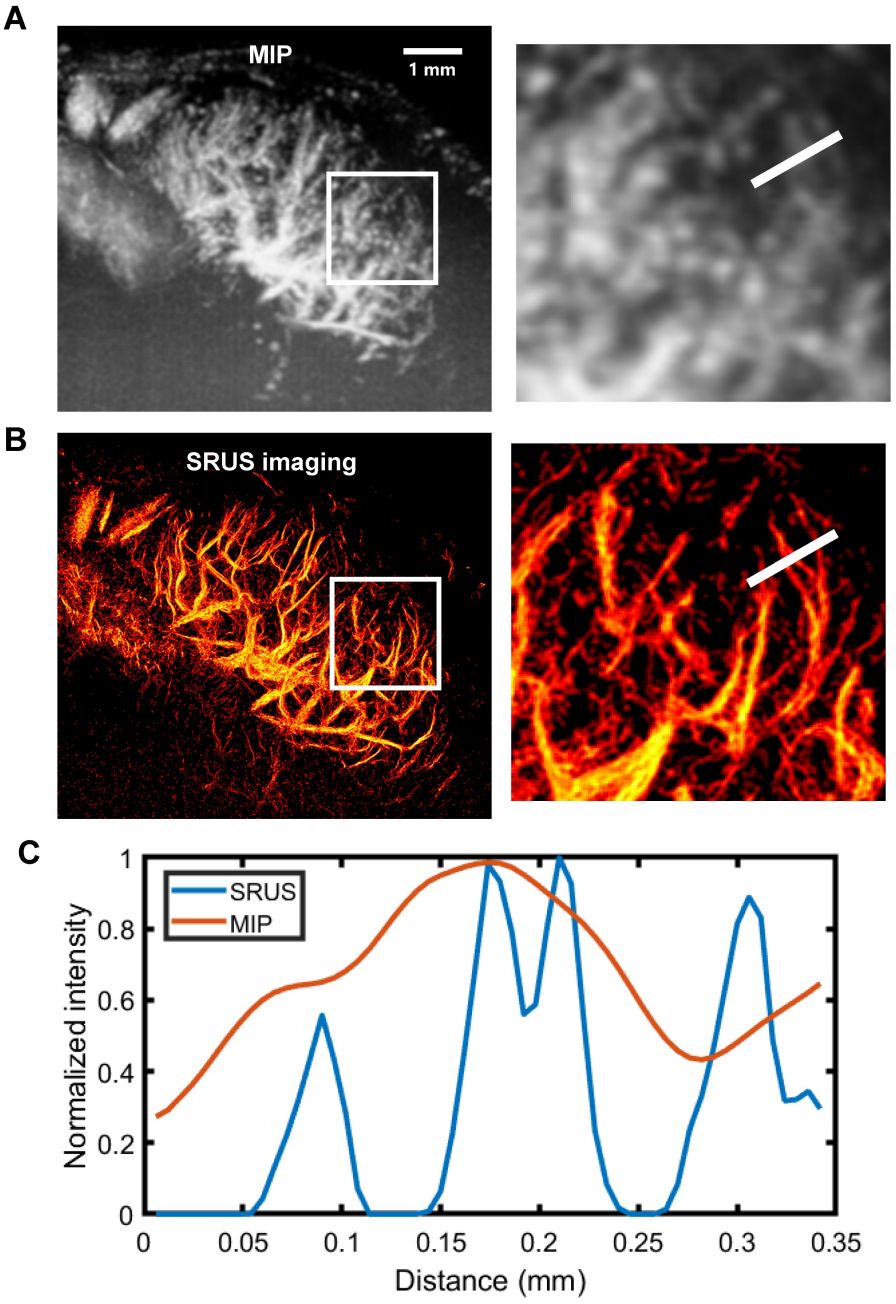
** SRUS imaging and spatial resolution quantification of a single lymph node cross-section.** (A) Maximum intensity projection (MIP) derived from a 1,500- contrast-enhanced ultrasound (CEUS) images. Local regions of interest (white rectangles) are enlarged for detailed comparison. (B) Reconstructed microvascular map using super-resolution ultrasound (SRUS) imaging. (C) Pixel intensity profile across the sampled region (white lines in A and B).

To evaluate the spatial resolution of SRUS, we performed a quantitative analysis using a representative 2D imaging plane of a mouse PALN (Figure 4). Comparison of the maximum intensity projection of CEUS image sequences (Figure 4A) with the corresponding SRUS image (Figure 4B) demonstrated that SRUS provides enhanced visualization of microvascular architecture. Notably, SRUS was able to resolve adjacent microvessels separated by only 31 μm (Figure 4C), confirming its superior spatial resolution over conventional CEUS.

**Figure 5:**
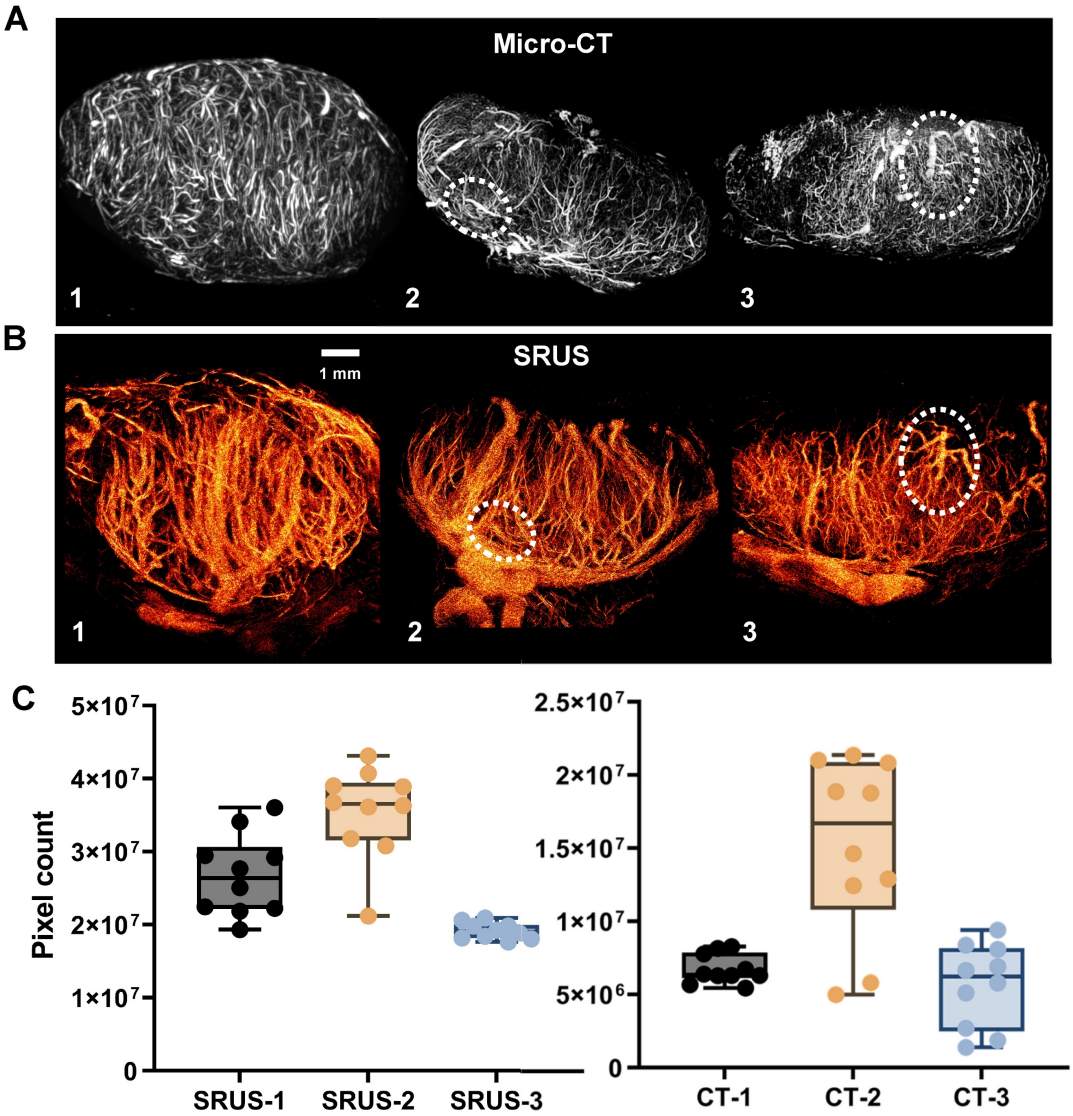
** Comparison of SRUS and micro-CT vascular reconstruction results.** (A) Representative summed intensity projections of *ex vivo* micro-CT images showing vascular architecture in three lymph nodes (LN 1-3). (B) Corresponding *in vivo* super-resolution ultrasound (SRUS) images acquired from the same lymph nodes. (C) Quantitative comparison of vascular pixel counts between micro-CT and SRUS for each lymph node. Vascular areas were calculated in pixel units from the central 10 imaging planes of each lymph node. Each dot represents an individual image slice. Left panel: SRUS pixel counts across LN 1-3; Right panel: corresponding micro-CT vascular pixel counts. Box plots indicate the median, interquartile range, and full data range. Statistical significance was evaluated using one-way ANOVA.

Figure 5 presents paired comparisons between SRUS-derived microvascular structures and gold-standard micro-CT angiograms of LNs. Video S1 provides SRUS imaging results across all image planes, while the 3D visualization of micro-CT data is shown in Video S2. Visual inspection of the corresponding images demonstrates comparable vascular morphological features between the two modalities (Figure 5A, B). Although full alignment of all vessel types is not achievable with the current SRUS resolution, the main vessels exhibited good structural correspondence with micro-CT (highlighted by white dashed circles in Figure 5). Additionally, we compared LN vascular area distributions derived from SRUS and micro-CT across different mice (Figure 5C). The results demonstrated good consistency in the vascular distribution trends between the two modalities, supporting the quantitative reliability of SRUS. Detailed statistical results has been provided in Tables S1 and S2.

**Figure 6:**
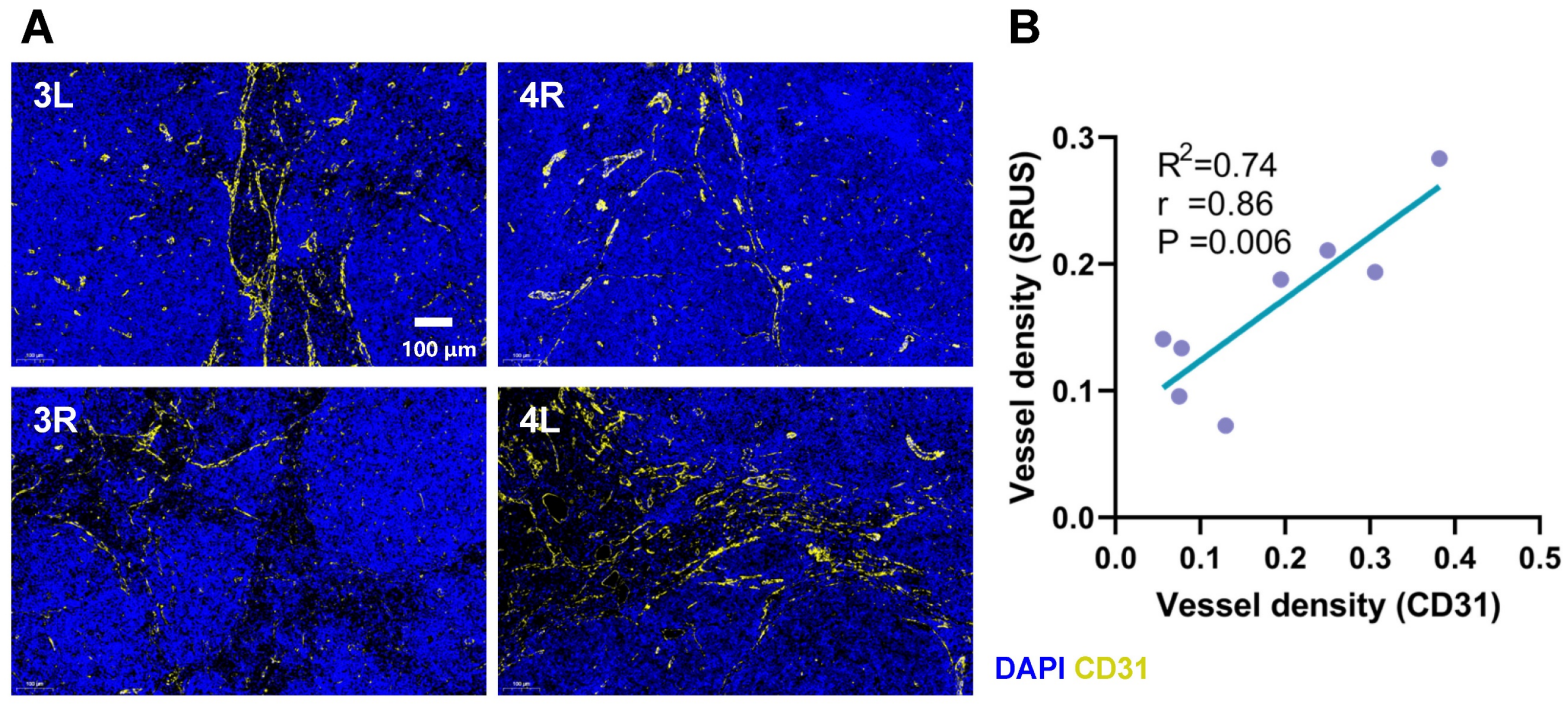
** Comparison between CD31 immunohistochemical staining and SRUS vascular quantification.** (A) Representative immunofluorescence images of lymph node sections stained with CD31 (yellow) and DAPI (blue) from four different specimens. 3L denotes the left PALN from Mouse 3. 4R denotes to the right PALN from Mouse 4. (B) Correlation analysis between vascular density measured by SRUS and histologically assessed CD31-positive vessel density. Each data point represents a single lymph node. Statistical analysis was performed using Pearson correlation with linear regression fitting.

We performed a comparative analysis between SRUS-derived vascular density measurements and histologically assessed vascular density based on CD31 immunofluorescence staining (Figure 6A). A strong correlation was observed between the two modalities (*r* = 0.86, p < 0.01, R² = 0.74; Figure 6B), supporting the reliability of SRUS for noninvasive assessment of vascular density within LNs.

Achieving 3D SRUS imaging of LN blood flow requires sustained MB stability. In our study, a full 3D acquisition typically exceeded 5 min due to respiratory gating, slice-by-slice acquisition, and intermittent data storage. Our long-lasting MBs, engineered with optimized shell composition and size distribution, provided consistent contrast enhancement throughout the entire scan following a single bolus injection. This prolonged *in vivo* stability is particularly advantageous for potential clinical translation, where extended imaging durations, repeated acquisitions, or larger volumetric coverage may be necessary.

### Correlation between SRUS-derived vascular parameters and lymph node physiological status

Beyond vascular structural imaging, SRUS also enables the evaluation of hemodynamic parameters within LN blood vessels. In contrast to conventional micro-CT, which provides high-resolution vascular images but lacks functional information, SRUS simultaneously offers hemodynamic insights of densely packed microvasculature (Figure S2A, B). This capability facilitates comprehensive evaluation of blood perfusion within LNs.

**Figure 7:**
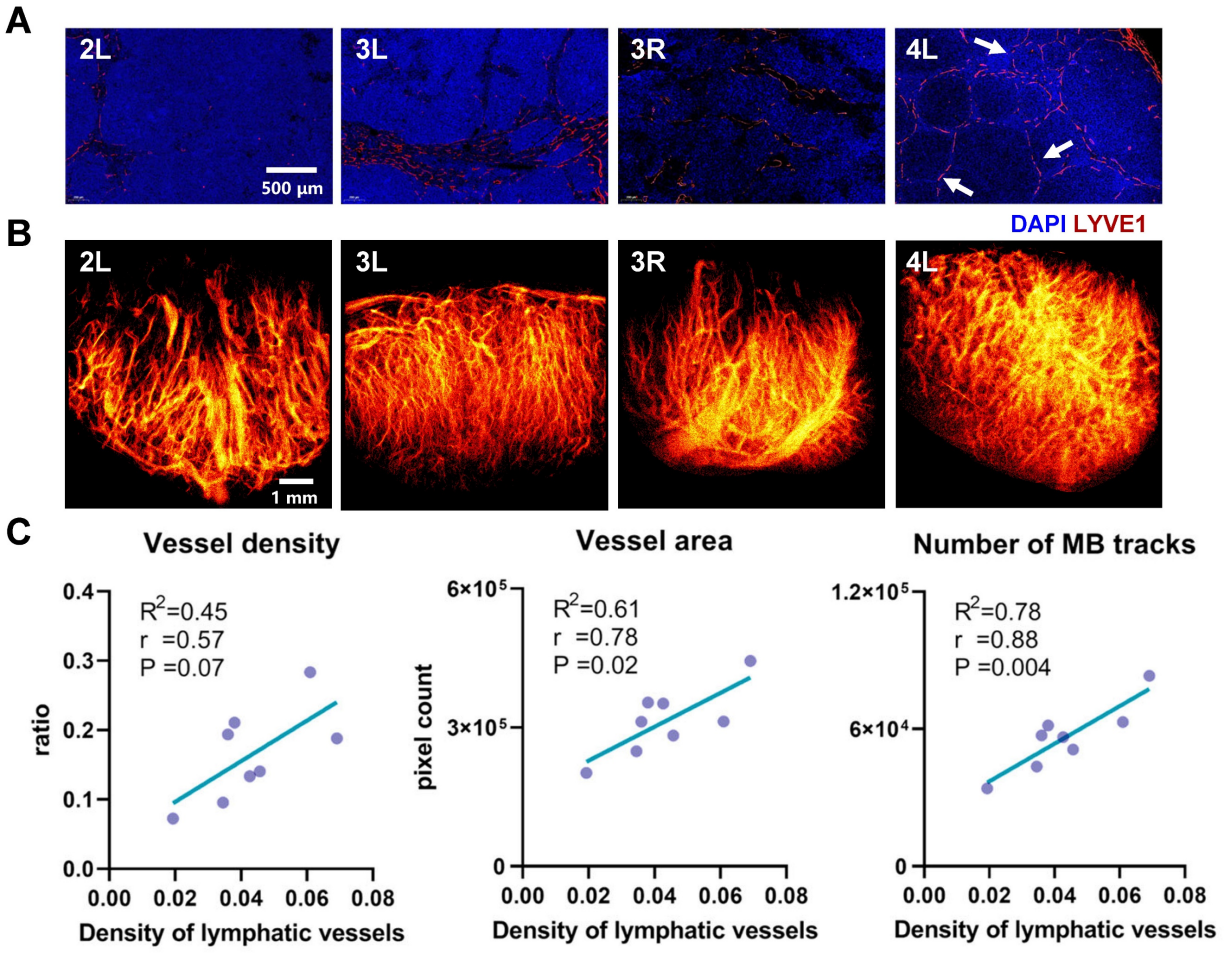
** Comparative analysis between histopathological features and SRUS-derived vascular parameters in murine lymph nodes.** (A) Immunofluorescence staining of lymphatic vessels. “4L” indicates the left PALN from Mouse 4. Blue: DAPI-stained nuclei; red: LYVE-1-positive lymphatic vessels. Arrows denote the locations of secondary follicles. (B) Corresponding super-resolution ultrasound (SRUS) images of the same lymph nodes. (C) Correlation analysis between histologically quantified lymphatic vessel density and SRUS-derived vascular parameters. Each data point represents an individual lymph node. Pearson correlation coefficients (*r*), p-values (P), and linear regression fits (R²: coefficient of determination) were calculated.

In this study, a systematic comparison was conducted between SRUS imaging and corresponding histopathological staining results across 8 LNs representing distinct developmental stages (Figure 7). Four representative LNs, exhibiting notable differences in immune status and vascular architecture, were selected and sequentially labeled based on increasing mouse age. The results revealed clear age-related changes in blood vessel density and immune activation within the LNs of MRL/lpr mice. Notably, the LN from Mouse 4 exhibited markedly increased vascular density and a higher number of secondary follicles (Figure 7A, 4L, white arrows) compared to those from Mice 2 and 3, indicative of enhanced immune activity, likely driven by B-cell activation and lymphangiogenic remodeling. The corresponding SRUS vascular image (Figure 7B) corroborates this observation, underscoring the capability of SRUS to capture immune-associated vascular remodeling within LNs.

To further investigate the relationship between blood and lymphatic vascular changes, we conducted a correlation analysis between SRUS-derived vascular parameters and lymphatic vessel density (Figure 7C and Figure S3). In this analysis, the proportion of LYVE1-positive area was used as a marker for LVD and an indicator of immune activation, a strategy supported by previous studies 37, 44, 45. Strong positive correlations were observed between lymphatic vessel density and both blood vessel area (*r* = 0.78, P = 0.02) and number of MB tracks (*r* = 0.88, P = 0.004), supporting the association between enhanced immune response and increased blood flow perfusion, consistent with previous findings 37, 46. Interestingly, average blood flow velocity did not exhibit a significant correlation with lymphatic vessel density, suggesting that lymphangiogenesis may not directly influence perfusion efficiency.

Beyond structural analysis, SRUS offers comprehensive hemodynamic characterization, including quantitative measurements of blood flow velocity within LN vasculature, which are unattainable with micro-CT or conventional histological techniques 47. While CEUS can assess LN immune status based on parameters such as vascular density and perfusion patterns, these metrics can be influenced by factors like LN enlargement or inflammation, potentially leading to false-positive or false-negative interpretations 48-50. In contrast, SRUS enables integrated multiparametric assessment of complex hemodynamics within LN vasculature, providing a promising non-invasive approach for the assessment of LN function and immune activation, without compromising patient safety.

### Association between blood vessel subtypes and lymph node physiological status

Given the inherent complexity and heterogeneity of hemodynamics in LN blood vessels 6, 37, 51, conventional analyses based on global vascular parameters may obscure critical regional variations in perfusion. Therefore, we developed an approach by categorizing vascular subtypes based on their distinct functional characteristics.

**Figure 8:**
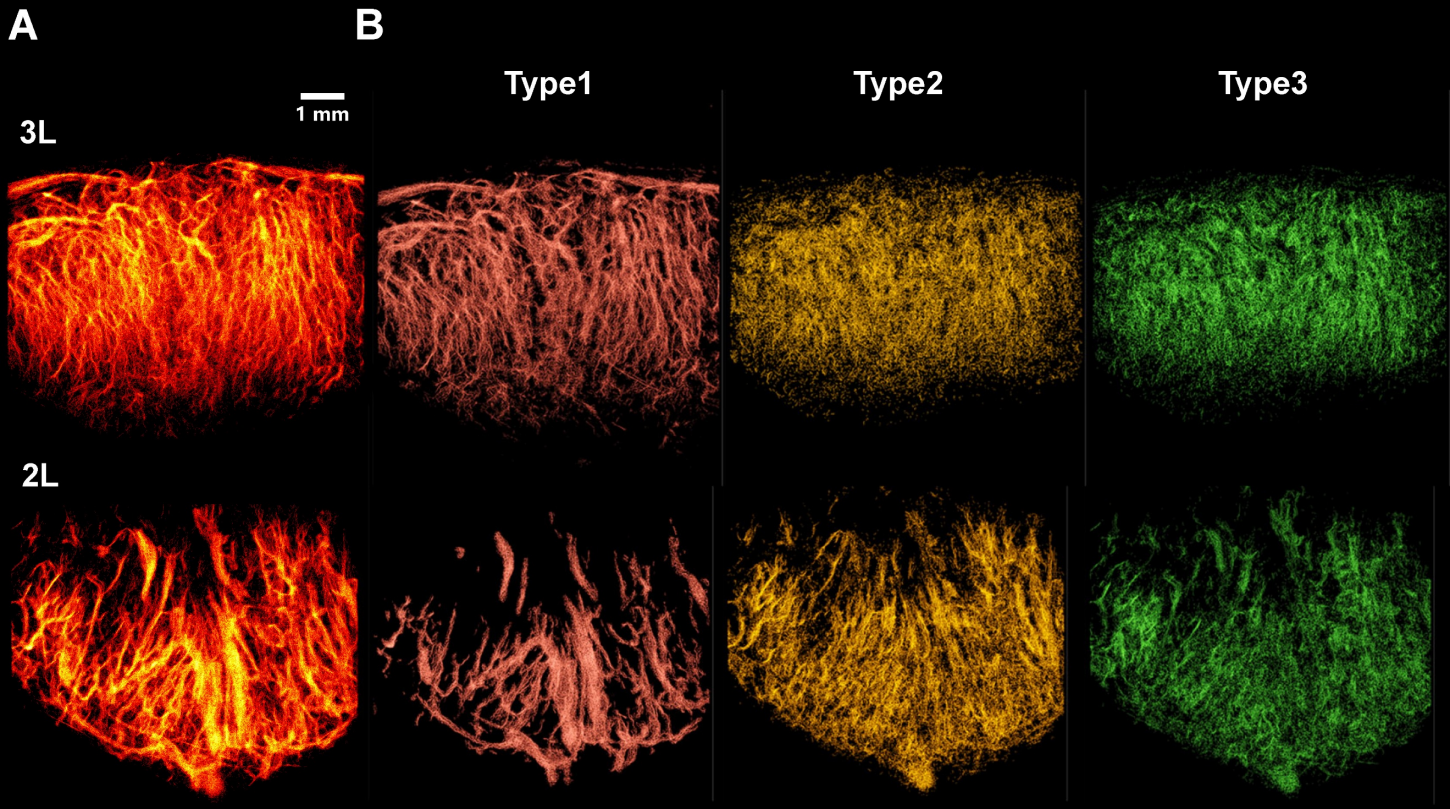
** Classification of vascular subtypes in murine lymph nodes based on microbubble dynamics using SRUS.** (A) Composite super-resolution ultrasound image showing microvascular reconstruction from multiplanar scans. 3L represents left PALN from Mouse 3. (B) Three distinct vascular subsets, including Type 1 (red, large-diameter vessels with high-speed blood flow), Type 2 (yellow, low-speed arterial branches), and Type 3 (green, venous flow).

Representative 3D SRUS vascular maps of LNs were selected for demonstration, as illustrated in Figure 8A. These maps reveal pronounced spatial heterogeneity in vascular architecture: large-diameter vessels are predominantly localized in the hilum region, whereas smaller vessel branches are primarily distributed throughout the peripheral cortex.

To categorize the different types of vessels, we applied a classification strategy based on flow velocity of MB tracks (Figure 8B), enabling separation of vascular subtypes based on their distinct hemodynamic profiles. As shown in Figure 8B, we successfully identified three major vascular subtypes: Type 1 (high-velocity, large-diameter blood flows concentrated in the hilar region, corresponding to major arteries and veins), Type 2 (low-speed arterial flows primarily present in branching vessels), and Type 3 (low-speed venous flows distributed throughout the venous branches). Due to poorly defined hilar anatomy in one lymph node, vascular inflow and outflow directions could not be confidently determined, and the sample was therefore excluded from further analysis.

**Figure 9:**
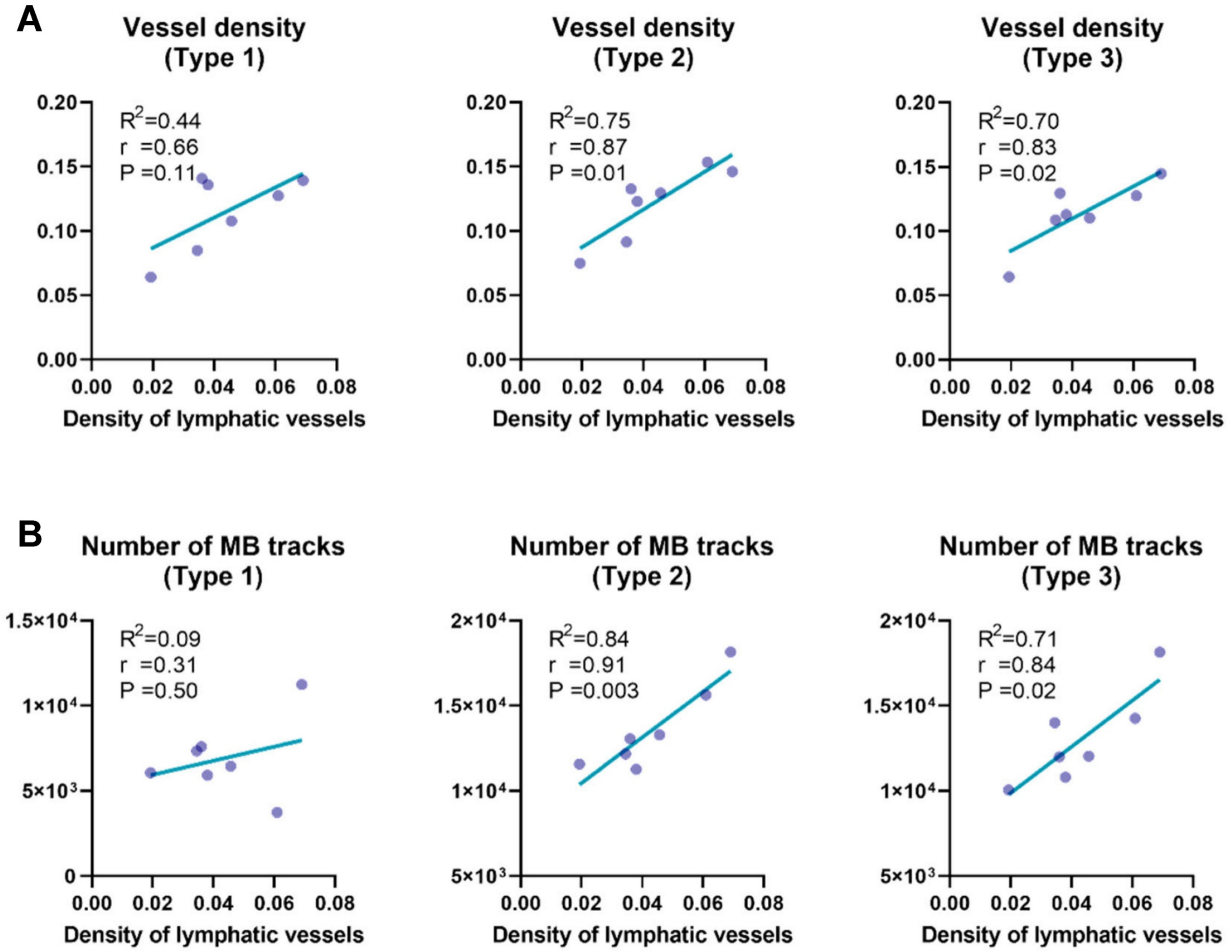
** Correlation analysis between lymphatic vessel density and vascular parameters across distinct vascular subtypes.** (A) Correlation between lymphatic vessel density, quantified by LYVE-1 immunofluorescence, and SRUS-derived vessel density across different vascular subgroups within lymph nodes. (B) Correlation between lymphatic vessel density and the number of microbubble (MB) tracks detected by super-resolution ultrasound (SRUS) imaging. Each data point represents one lymph node. Statistical analysis was performed using Pearson correlation with linear regression. Reported values include the correlation coefficient (*r*), coefficient of determination (R²), and p-value (P).

Quantitative analysis of vessel subtypes (Figure 9) revealed that microvascular parameters associated with small vessels (i.e., Type 2 and Type 3) exhibited stronger correlations with lymphatic vessel density, compared to Type 1 vessels. Specifically, vessel density (Figure 9A; *r* = 0.87 for Type 2, *r* = 0.83 for Type 3) and the number of MB tracks (Figure 9B; *r* = 0.91 for Type 2, *r* = 0.84 for Type 3) were highly correlated with lymphatic vessel density, underscoring the critical role of microcirculatory remodeling in LNs. Our results provide compelling evidence supporting previous findings that inflammatory lymphadenopathy can induce up to threefold volumetric expansion, accompanied by marked angiogenesis within capillary endothelial subpopulations 6, 46, 52-54.

Despite the clinical relevance of LN status, there are currently no established non-invasive imaging biomarkers capable of reliably evaluating LN activity. This presents significant challenges in managing both malignant and inflammatory lymphadenopathies. Our results demonstrate that SRUS imaging can fill this gap by offering high-resolution visualization of hemodynamics in LN blood vessels. The microvascular parameters derived from SRUS can serve as quantitative biomarkers of LN immune activity, thus providing a new framework for precise diagnostic and prognostic assessment.

Emerging evidence suggests that characteristic microvascular patterns within axillary LNs may serve as reliable indicators of metastatic potential, particularly in cases where conventional ultrasound yields inconclusive results 55. Metastatic LNs typically demonstrate significantly increased vascular density accompanied by distinctive hemodynamic alterations 55, such as changes in resistance index (RI) and pulsatility index (PI) 56, 57. By detecting subtle changes in microvascular density and flow velocity within axillary lymph nodes, SRUS have demonstrated its potential in predicting metastatic sentinel lymph nodes (SLNs) in breast cancer patients 58.

Compared to 19 and 58, the present work focuses on the relationship between microvascular features and immune activity within LNs, aiming to assess overall immune activation status. By integrating a novel lipid-based long-lasting contrast agent with 3D SRUS imaging, we introduce a vascular subtyping strategy based on hemodynamic parameters of blood flow. To our knowledge, this is the first study to systematically correlate microvascular features with immune functional states in LNs using SRUS. As a non-invasive, real-time, and high-resolution imaging modality, this framework provides a novel approach for individualized immunotherapy evaluation and holds promise for clinical translation in LN-related disease management.

In this study, the characteristic lymphoid hyperplasia, immune activation, and extensive microvascular remodeling of MRL/lpr model provide a platform for investigating the relationship between LN blood vessels and immune status. We believe the multiparametric imaging capabilities of SRUS demonstrate strong promise for broader applicability. These features can be extended to various clinical scenarios, including SLN monitoring in cancer, evaluation of responses to immunotherapy, and diagnosis of infectious or inflammatory conditions.

## Conclusion

This study addresses the critical need for noninvasive imaging modalities capable of functional characterization of lymph node activity. Although SRUS offers unprecedented capabilities in visualizing complex microvessels, challenges remain in delineating the complex functional interplay between the vascular and lymphatic systems. Based on our long-lasting microbubbles, SuperVue-MB, we achieved three-dimensional multiparametric mapping of lymph node blood vessels, and developed a novel vascular subtyping strategy that systematically correlates these hemodynamic patterns of blood vessels with immune functional states. This bridges the gap between microvascular imaging biomarkers and pathophysiological progression, offering a powerful tool for comprehensive evaluation of lymph node associated diseases.

## Supplementary Material

Supplementary figures, tables, and videos.

## Abbreviations

MB: microbubble
SRUS: super-resolution ultrasound imaging
CEUS: contrast-enhanced ultrasound
ULM: ultrasound localization microscopy
LN: lymph node
LVD: lymphatic vessel density
PALN: proper axillary lymph node
