# Supplementary material for: Long-lasting microbubble-enhanced super-resolution ultrasound imaging unveils lymphatic activity in lymph node
Source: Theranostics. 2025 Aug 11;15(17):8857–72. doi: 10.7150/thno.117971 (PMC12439261; doi:10.7150/thno.117971)
Supplement: Supplementary file 1 — Supplementary figures, tables, and videos. [file thnov15p8857s1.zip › SupplementaryMaterial.pdf]

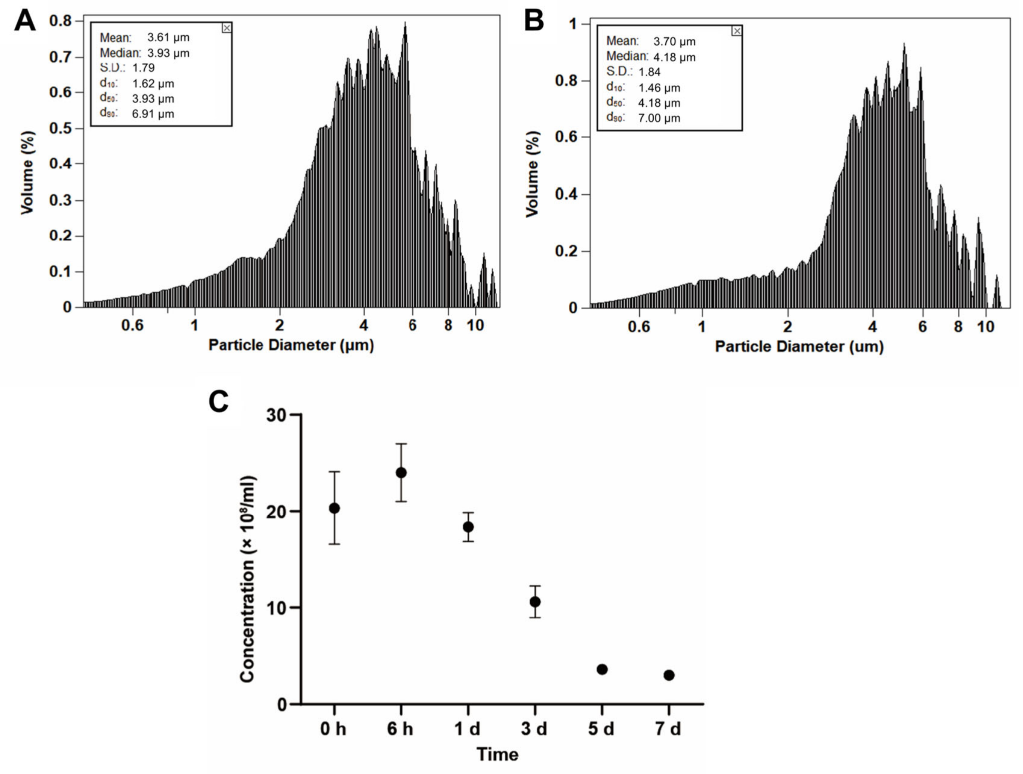

Figure S1. Stability characterization of microbubbles. (A, B) Particle size distribution of lyophilized microbubbles upon reconstitution at Day 0 (A) and Day 180 (B). (C) Concentration variation of reconstituted microbubbles over time.

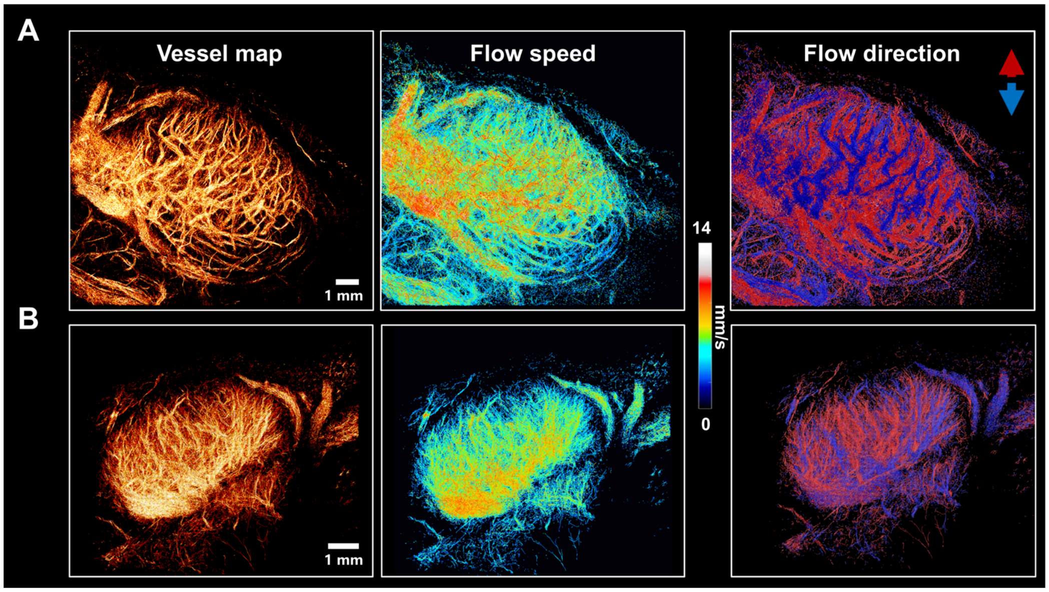

Figure S2. SRUS imaging of representative lymph nodes. (A, B) Super-resolution ultrasound (SRUS)-based microvascular reconstructions reveal densely organized vascular networks within lymph nodes across multiple imaging planes. Compared to micro-CT imaging (gray-scale, Video S2), which provides only anatomical structure, SRUS offers visualization of hemodynamic parameters, including flow speed (color-coded) and flow direction (indicated by arrows).

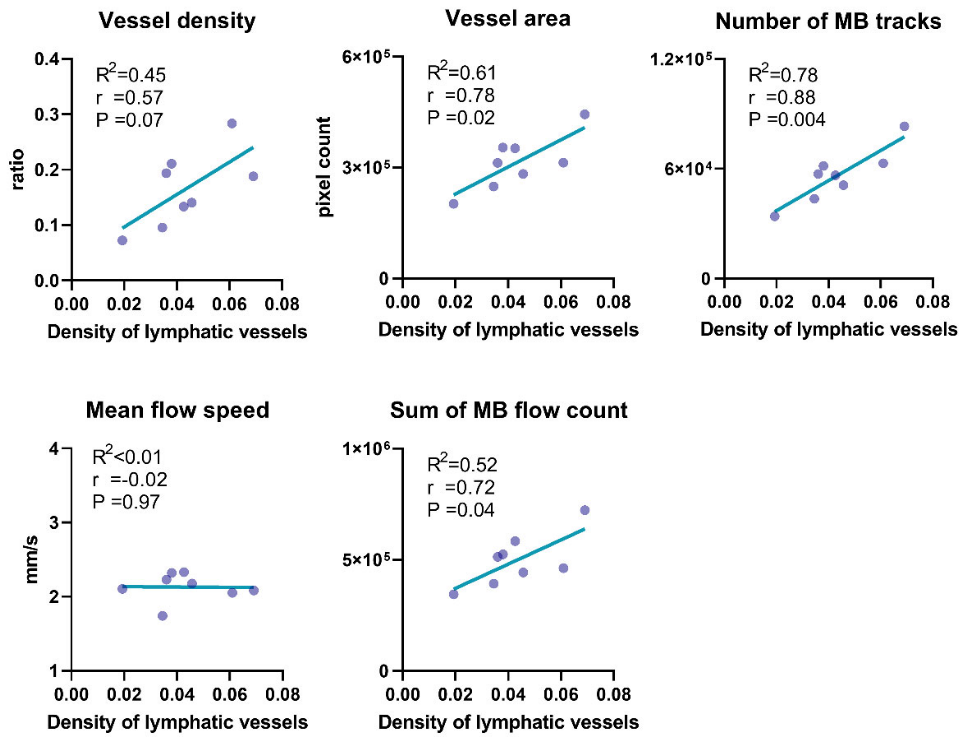

Figure S3. Correlation analysis between histologically quantified lymphatic vessel density and SRUS-derived vascular parameters. Scatter plots illustrate the relationship between lymphatic vessel density measured from histological sections and corresponding vascular parameters obtained via super-resolution ultrasound (SRUS) imaging. Each data point represents an individual lymph node. Statistical analysis was performed using Pearson correlation with linear regression. Reported metrics include the correlation coefficient (*r*), coefficient of determination (R^2), and p-value (P).

Table S1. The Tukey's multiple comparisons test of SRUS results

Pairs	Mean Diff.	95.00% CI of diff.	Below threshold?	Adjusted P Value
SRUS-1 vs. SRUS-2	-8723474	-14115523 to -3331424	Yes	0.0012
SRUS-1 vs. SRUS-3	7581303	2189254 to 12973352	Yes	0.0047
SRUS-2 vs. SRUS-3	16304777	10912727 to 21696826	Yes	<0.0001

Table S2. The Tukey's multiple comparisons test of micro-CT results

Pairs	Mean Diff.	95.00% CI of diff.	Below threshold?	Adjusted P Value
CT-1 vs. CT-2	-8432238	-12784200 to -4080276	Yes	0.0001
CT-1 vs. CT-3	1098668	-3253295 to 5450630	No	0.8073
CT-2 vs. CT-3	9530906	5178943 to 13882868	Yes	<0.0001

Video S1. Super-resolution ultrasound (SRUS) imaging of microvascular structures within the lymph node of an MRL/lpr mouse. The video presents reconstructed microvascular images of sequential 2D imaging planes.

Video S2. Three-dimensional rotational view of micro-CT reconstruction of the lymph node microvasculature in an MRL/lpr mouse.
